# Physical activity and risk of comorbidities in patients with chronic obstructive pulmonary disease: a cohort study

**DOI:** 10.1038/s41533-017-0034-x

**Published:** 2017-05-18

**Authors:** Tsung Yu, Gerben ter Riet, Milo A. Puhan, Anja Frei

**Affiliations:** 1 Epidemiology, Biostatistics, and Prevention Institute, University of Zurich, Hirschengraben 84, CH-8001 Zurich, Switzerland; 2 Department of Public Health, China Medical University and Big Data Center, China Medical University Hospital, Taichung, Taiwan; 3 Department of General Practice, Academic Medical Center, University of Amsterdam, Amsterdam, The Netherlands

## Abstract

Multi-morbidity is common in patients with chronic obstructive pulmonary disease and low levels of physical activity are hypothesized to be an important risk factor. The current study aimed to assess the longitudinal association between physical activity and risk of seven categories of comorbidity in chronic obstructive pulmonary disease patients. The study included 409 patients from primary care practice in the Netherlands and Switzerland. We assessed physical activity using the Longitudinal Ageing Study Amsterdam Physical Activity Questionnaire at baseline and followed patients for up to 5 years. During follow-up, patients reported their comorbidities (cardiovascular, neurological, endocrine, musculoskeletal, malignant, and infectious diseases) and completed the Hospital Anxiety and Depression Scale questionnaire for mental health assessment. We implemented multinomial logistic regression (an approximation to discrete time survival model using death as a competing risk) for our analysis. Study results did not suggest a statistically significant association of baseline physical activity with the development of seven categories of comorbidity. However, when we focused on depression and anxiety symptoms, we found that higher levels of physical activity at baseline were associated with a lower risk for depression (adjusted hazard ratio, 0.85; 0.75–0.95; *p* = 0.005) and anxiety (adjusted hazard ratio, 0.89; 0.79–1.00; *p* = 0.045). In chronic obstructive pulmonary disease patients, those with high physical activity are less likely to develop depression or anxiety symptoms over time. Increasing physical activity in chronic obstructive pulmonary disease patients may be an approach for testing to lower the burden from incident depression and anxiety.

## Chronic lung disease: stay active to avoid depression and anxiety

Patients with chronic lung disease who stay physically active could reduce their chances of depression and anxiety. Milo Puhan at the University of Zurich, Switzerland, and co-workers assessed the association between physical activity and the risk of developing various co-existing diseases in 409 patients with chronic obstructive pulmonary disease (COPD). Co-morbidities such as cardiovascular diseases, diabetes and depression are prevalent in patients with COPD, but the reasons why are not clear. Puhan’s team assessed patients’ activity levels using an existing questionnaire, and administered another questionnaire to assess mental health. They followed the cohort for 5 years. Results indicated weak associations between physical activity levels and most physical illnesses, but there were significant links between higher levels of physical activity and a reduced risk of depression and anxiety. The results could inform novel COPD treatment programs.

## Introduction

The clinical manifestations of chronic obstructive pulmonary disease (COPD) are diverse. Five decades ago, the “pink puffer” and the “blue bloater” were introduced as a way to show distinct combinations of clinical manifestations, which included cardiovascular comorbidity.^1^ More recently, researchers have attempted to identify phenotypes of COPD and also pointed out the prominent role of comorbidities.^2^ Comorbidities such as cardiovascular diseases, diabetes mellitus, depression, and musculoskeletal disorders are more prevalent in COPD patients than in sex- and age-matched healthy individuals.^3^ Besides reducing patients’ health-related quality of life,^4^ comorbidities are associated with an increased risk of all-cause hospital admission and death,^5^ and they contribute to more health-care use and cost.^6^

Low physical activity (PA) is an important feature of COPD patients and has received much attention in research recently.^7–9^ Consistent evidence suggests that low PA levels are associated with death and COPD exacerbations,^7^ and are hypothesized to be an important risk factor for multi-morbidity in COPD patients.^10–12^ Some biological mechanisms have been proposed to explain these associations. For example, low PA levels may adversely affect lipid lipoprotein profiles, glucose homeostasis, insulin sensitivity, and inflammation status, which together increase the risk for developing chronic diseases in these patients.^13^

However, most studies on the association of PA with comorbidity in COPD were cross-sectional. For instance, Remoortel and colleagues^11^ recently demonstrated in a population-based study that smoking and physical inactivity were strongly associated with the presence of comorbidity in pre-clinical COPD patients. Since longitudinal evidence is scarce, the issue of reverse causation may severely limit the validity of some of these findings.^7^

The aim of the current study was to assess the longitudinal association of PA with the incidence of comorbidities. We studied this in a 5-year prospective follow-up study of COPD patients including seven categories of comorbidity, that is, cardiovascular, neurological, endocrine, musculoskeletal, mental, malignant, and infectious diseases.

## Results

### Description of study subjects

The study recruited 258 patients from the Netherlands and 151 patients from Switzerland, respectively. Baseline characteristics of patients are shown in Table 1 and more details were reported previously.^14, 15^ The median of the Longitudinal Ageing Study Amsterdam Physical Activity Questionnaire (LAPAQ) score that is indicative of types of PA and weighted by intensity was 11 (interquartile range 8.5–15). The median number of visits after which an additional comorbidity was identified was three (equivalent to 1.5 years after baseline).

**Table 1:** Baseline characteristics of study patients

Characteristics	Number of patients: 409
Nationality	
Switzerland, *n* (%)	151 (37%)
The Netherlands, *n* (%)	258 (63%)
Sex	
Male, *n* (%)	233 (57%)
Female, *n* (%)	176 (43%)
Mean (SD) age, years	67.3 (10.0)
Mean (SD) BMI, kg/m^2^	26.2 (5.2)
Current smokers, *n* (%)	156 (39%)
Mean (SD) smoking pack years	44.1 (27.8)
Mean (SD) FEV_1_, % of predicted	55.5 (16.6)
Mean (SD) FEV_1_, liters	1.5 (0.6)
mMRC dyspnea scale	
0–1, *n* (%)	226 (55%)
≥2, *n* (%)	183 (45%)
Exacerbations in the previous year	
None, *n* (%)	273 (67%)
≥1, *n* (%)	136 (33%)
Types of physical activity	
Walking, *n* (%)	360 (88%)
General bicycling, *n* (%)	102 (25%)
Gardening, *n* (%)	124 (30%)
Sports, *n* (%)	172 (42%)
Light household activities, *n* (%)	367 (90%)
Heavy household activities, *n* (%)	238 (58%)

*BMI* body mass index, *FEV1* forced expiratory volume in 1 s, *mMRC* Modified British Medical Research Council, *SD* standard deviation

Table 2 shows the prevalence of seven categories of comorbidity at study baseline.

**Table 2:** Prevalence of comorbidities at study baseline (*N* = 409)

Comorbidity	*n* (%)
Cardiovascular diseases	149 (36%)
Symptomatic heart diseases (coronary heart diseases or myocardial infarction or angina pectoris or heart failure)	80 (20%)
Other cardiovascular diseases	97 (24%)
Neurological disorders	59 (14%)
Cerebrovascular accident	37 (9%)
Other neurological disorders	28 (7%)
Endocrine disorders	97 (24%)
Diabetes mellitus	63 (15%)
Other endocrine disorders	46 (11%)
Musculoskeletal disorders	146 (36%)
Rheumatoid arthritis	15 (4%)
Arthrosis	51 (12%)
Other musculoskeletal disorders	112 (27%)
Mental disorders (anxiety or depression)	66 (16%)
Anxiety	44 (11%)
Depression	41 (10%)
Cancers	58 (14%)
Infectious diseases	20 (5%)

### Association of PA with incident comorbidities

**Fig. 1:**
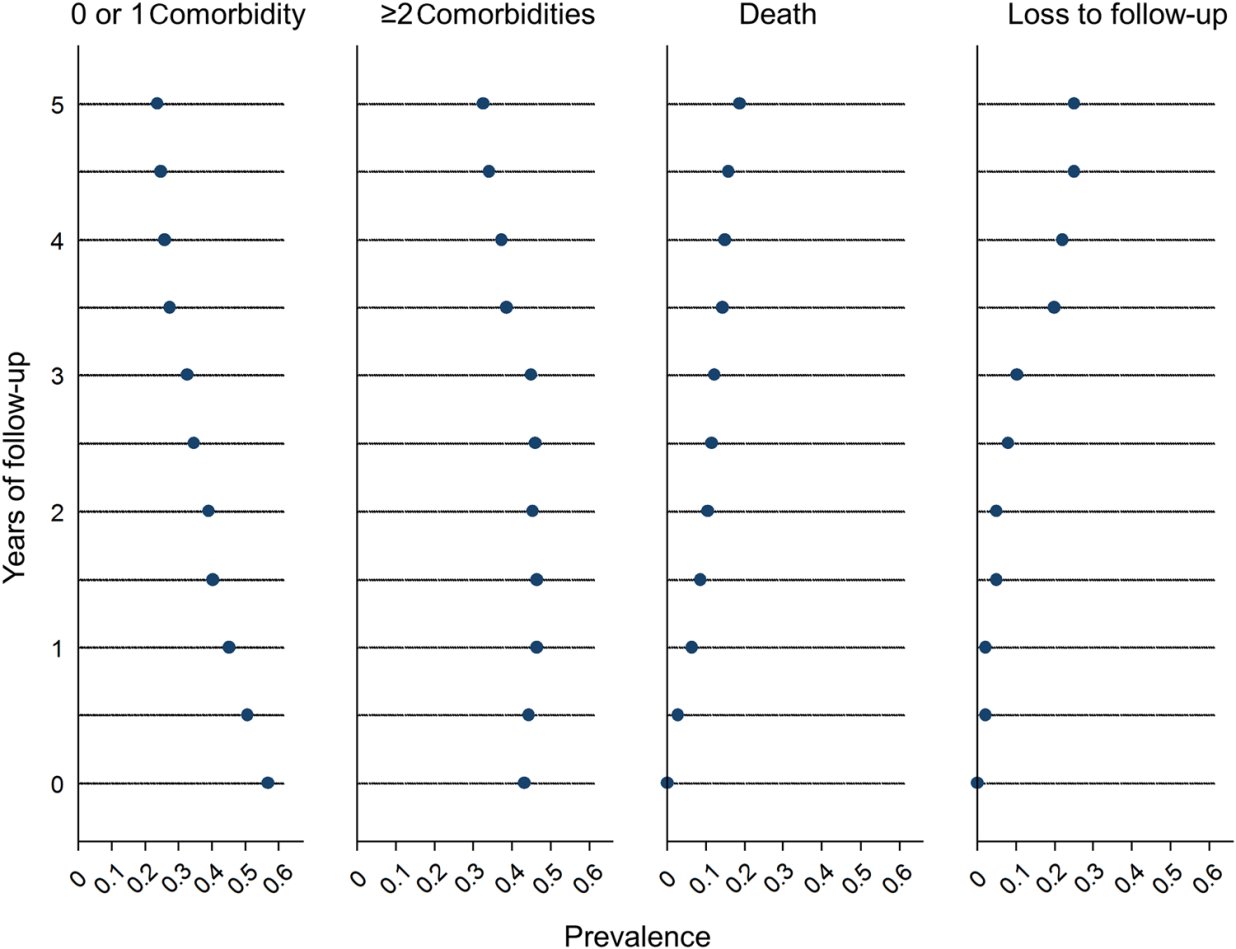
Distribution of patients with 0 or 1 category of comorbidities, ≥2 categories of comorbidities, and patients who were dead or lost to follow-up over time (*n* = 409). Seven categories of comorbidities were included. At each visit, we recorded if the patients had been diagnosed with cardiovascular diseases, neurological disorders, endocrine disorders, musculoskeletal disorders, and cancers and we also recorded if the patients had ever reported having infections and mental disorders (anxiety or depression) after being enrolled in the study. Over time, patients in the groups “0 or 1 comorbidity” or “≥2 comorbidities” can move to the group “death” or “loss to follow-up”. Also, patients in the group “0 or 1 comorbidity” can move to the group “≥2 comorbidities”

In Fig. 1 we classified the patients at every visit into those with 0 or 1 category of comorbidities, those with  ≥ 2 categories of comorbidities, and those who were dead or lost to follow-up. As expected, the proportion of patients with 0 or 1 comorbidity decreased over time. During study follow-up, the number of new cases of cardiovascular diseases was 24, of neurological disorders 24, of endocrine disorders 5, of musculoskeletal disorders 34, of mental disorders 92, of cancers 13, and of infectious diseases 13.

Overall, the risk of developing any additional comorbidity over time was 7% lower for a 2.5-point increase in the LAPAQ score at baseline (unadjusted hazard ratio = 0.93; 95% confidence interval (CI): 0.87–0.99; *p* = 0.03). The corresponding figures (unadjusted hazard ratios) by category of comorbidity were 0.96 (0.80–1.15; *p* = 0.63) for cardiovascular diseases, 0.97 (0.80–1.16; *p* = 0.71) for neurological disorders, 0.82 (0.54–1.26; *p* = 0.37) for endocrine disorders, 0.88 (0.76–1.03; *p* = 0.12) for musculoskeletal disorders, 0.90 (0.82–0.99; *p* = 0.04) for mental disorders, 0.80 (0.61–1.03; *p* = 0.08) for cancers and 0.86 (0.72–1.04; *p* = 0.12) for infectious diseases.

**Fig. 2:**
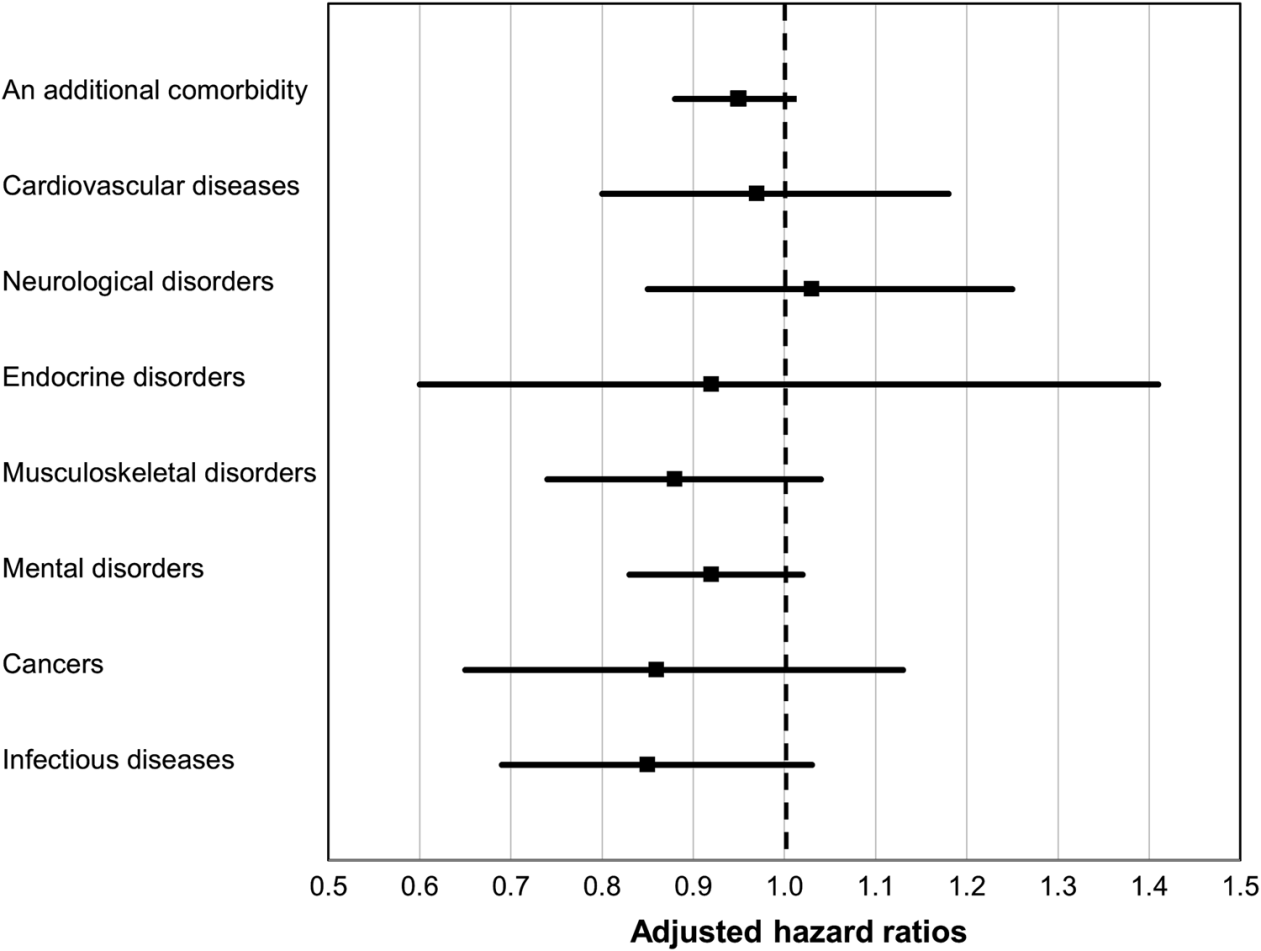
Associations of physical activity with incident comorbidities. The relative-risk ratios estimated from multinomial logistic regression models (competing risk model) were interpreted as hazard ratios. Age, sex, body mass index, smoking pack-years, and forced expiratory volume in 1 s (liters) were included as covariates in the model

We also computed adjusted hazard ratios for risk of comorbidities overall and by the seven categories, with regard to a 2.5-point increase in the LAPAQ score (Fig. 2). The adjusted hazard ratios were 0.95 (95% CI: 0.88–1.01; *p* = 0.12) for developing an additional comorbidity, 0.97 (95% CI: 0.80–1.18; *p* = 0.77) for cardiovascular diseases, 1.03 (0.85–1.25; *p* = 0.73) for neurological disorders, 0.92 (0.60–1.41; *p* = 0.71) for endocrine disorders, 0.88 (0.74–1.04; *p* = 0.12) for musculoskeletal disorders, 0.92 (0.83–1.02; *p* = 0.13) for mental disorders, 0.86 (0.65–1.13; *p* = 0.27) for cancers and 0.85 (0.69–1.03; *p* = 0.09) for infectious diseases.

We did post hoc analysis on the association of PA with the incidence of depression and anxiety in particular because the previous analyses suggested that there may be an association of PA with mental disorders. Also, compared to other comorbidities, there were many more cases and thus more power for analysis, with both depression (79 cases) and anxiety (80 cases). Results from the competing risk model are shown in Table 3. The adjusted hazard ratios for depression and anxiety were 0.85 (0.75–0.95; *p* = 0.005) and 0.89 (0.79–1.00; *p* = 0.045), respectively.

**Table 3:** Physical activity and risk of depression and anxiety in competing risk model (multinomial logistic regression)

Risk factors	Model 1: Depression		Model 2: Anxiety	
	HR (95% CI)	*p* value	HR (95% CI)	*p* value
Physical activity (LAPAQ score), 2.5 points difference	0.85 (0.75–0.95)	0.005	0.89 (0.79–1.00)	0.045
Age in years	0.98 (0.96–1.01)	0.12	1.00 (0.97–1.02)	0.75
Smoking pack-year	1.01 (1.00–1.02)	0.01	1.01 (1.00–1.01)	0.18
BMI	1.01 (0.97–1.06)	0.62	0.99 (0.95–1.04)	0.75
Sex (male = reference)	0.87 (0.52–1.44)	0.58	1.45 (0.88–2.40)	0.15
FEV1 in liters	0.57 (0.36–0.91)	0.02	0.91 (0.57–1.46)	0.70

The relative-risk ratios estimated from multinomial logistic regression models (competing risk model) were interpreted as hazard ratios

*BMI* body mass index, *FEV1* forced expiratory volume in 1 s, *HR* hazard ratio, *LAPAQ* Longitudinal Ageing Study Amsterdam Physical Activity Questionnaire

Results of sensitivity analyses with the average of PA scores over time as the exposure were similar to results of main analyses, except that the effect on anxiety became statistically non-significant (e-Appendix).

## Discussion

The current study investigated the association of PA with the risk of seven categories of comorbidity in patients with COPD. Using data from a 5-year longitudinal study, we found that higher levels of PA are associated with a lower risk of developing clinically relevant depression and possibly anxiety symptoms, for which we considered death as a competing event. However, for the other comorbidities that were examined here, there was inconclusive to weak evidence to suggest a statistically significant association of PA with incident risk of comorbidities.

Our study, in line with other previous research,^3^ found a high prevalence of comorbidities in patients with COPD. Many investigations^5, 16^ quantified the impact of comorbidities on patient’s health status and on their risk of death. For example, we reported previously that comorbidities including depression, anxiety, and peripheral artery disease had a large impact on patient’s health status.^16^ In another study, investigators included 12 comorbidities such as lung cancer, breast cancer, and anxiety to construct an index that aims to assess the impact of comorbidities on patient’s risk of death.^5^ To alleviate the impact of comorbidities on patient’s health as well as to develop and optimize treatment strategies for COPD, studying the modifiable risk factors for comorbidities, such as physical inactivity, would thus be extremely important.

A primary strength of this analysis is the use of data from a prospective cohort study. Previous research was heavily based on prevalent cases of comorbidities so there was a high likelihood that lower levels of PA were in fact a result of comorbidities. For example, a recent prospective study showed that depression symptoms are associated with reduced PA measured 6 months later in patients with COPD.^17^ Our longitudinal study design permitted us, firstly, to clarify the temporal relationship between PA and comorbidities. Secondly, we captured the incident cases of comorbidities owing to the prospective follow-up design. Thirdly, with mortality data, we can conduct thorough analysis that accounted for death as a competing risk against comorbidities. Although we did not observe a statistically significant association for most comorbidities examined, the findings suggest, in both unadjusted and adjusted analyses, that higher PA levels may be associated with a lower risk of most comorbidities.

There may be misclassification of our exposure and outcome. In particular, our assessment of PA was performed by patient self-report, which is prone to recall and response bias^18^ and may under- or over-estimate PA. Furthermore, the comorbidities were assessed based on patients’ self-report every half-year, instead of more objective physiological measurements or clinician’s diagnosis. It is difficult to assess the net impact of these various sources of potential misclassifications on our findings. Given that substantial epidemiological evidence has suggested the health benefits of PA for preventing chronic diseases in general population and subpopulations,^19, 20^ we believe, on the whole, attenuation of a protective effect of PA against developing comorbidities would be most likely. Often, non-differential misclassification of exposures or outcomes will attenuate the true association, which is probably the case here.

We aggregated some comorbidities (e.g., coronary heart diseases, myocardial infarction, etc. into cardiovascular diseases) to avoid potential diagnostic overlap and to increase statistical power. This aggregation, however, implies that we assumed identical pathophysiologic mechanisms underlying the role of physical inactivity in causing the various diseases within a cluster. Such an assumption may be reasonable since studies have shown that PA improves many physiological measures such as for cardiorespiratory and muscular fitness, bone health, and cognitive function.^19, 20^ In a recent pooled analysis of 1.44 million participants from cohort studies,^21^ higher levels of leisure-time PA were even found to be associated with lower incidences of 13 types of cancer. We do not know to what extent our aggregation of outcomes impaired the study validity. Studies that examine the benefits of PA in each type of chronic disease are needed in order to test the assumptions we made.

Another weakness of the study includes that our study sample did not include COPD patients at the early stage (i.e., Global Initiative for Chronic Obstructive Lung Disease (GOLD) stage 1), while it has been shown that reduced PA is a critical feature of these patients.^22^ Our assessment of patients’ PA was at more advanced stages of the disease, so we may not have captured the exposure (PA levels) at the most relevant time points, assuming that early decrease in PA is important to the development of comorbidities. Finally, we modeled PA levels as a continuous variable. It is likely that the associations between PA and risk of some comorbidities are not linear (on logit scale) or a threshold value of PA exists; however, to test for this, a much larger sample size (number of events in survival analysis) is needed.

### Implications

Mental disorders are more common in patients with COPD than in the general population,^23^ and depression and anxiety have been shown to increase the risk of COPD exacerbations, and possibly death.^23^ Searching for effective interventions to cope with depression and anxiety is thereby of great importance to clinicians who manage COPD patients. The negative association between PA and depression or anxiety in the general population has been widely suggested in previous research, such as in the National Comorbidity Survey in the United States.^24^ Our study showed that this may also apply to COPD as we found more physically active patients (with higher PA levels equivalent to 2.5 metabolic equivalent tasks (METs), e.g., those who report walking outside vs. those who do not) had 15% and 11% lower risks of developing depression and anxiety, respectively, compared to physically inactive patients. Most existing pulmonary rehabilitation programs for COPD patients comprise exercise as the main component. There is trial evidence that suggests pulmonary rehabilitation reduces symptoms of existing depression and anxiety^25^ but it is unclear if such interventions also lower the risk of incident depression and anxiety. In the future, it would be helpful for patients to have strategies to increase and maintain exercise capacity and PA beyond rehabilitation programs by using a wide range of interventions targeted at individuals or community in patient’s daily life.

## Materials and methods

### Subjects

The current study included 409 patients with COPD from the International collaborative effort on chronic obstructive lung disease: exacerbation risk index cohorts (ICE COLD ERIC) study.^14, 15^ Patients with GOLD stage 2–4 who were aged ≥ 40 years, who had been free of exacerbations for ≥4 weeks were recruited in the Netherlands and Switzerland between 2008 and 2009. Patients considered having  ≤ 12 months of life expectancy and having serious mental disorders were excluded. All patients provided their informed consent and the study was approved by the corresponding ethics boards.

### Assessment of PA

We used the LAPAQ to assess patients’ PA levels.^26^ At baseline, patients were asked to indicate the frequency and duration of six types of PA they had performed during the previous 2 weeks. A LAPAQ score that integrates the time spent on each PA (minutes per day in the previous 2 weeks) and the intensity of each PA, informed by the METs,^27^ can be generated. However, and as described previously,^28^ we constructed a new LAPAQ score omitting information on frequency and duration, where we found missing or implausible values. We used patients’ responses to the types of PA only and assigned weights to each PA according to the METs: 2.5 for outside walking, 4.0 for bicycling, 4.0 for gardening, 6.0 for sports, 2.5 for light household activities and 4.0 for heavy household activities. Thus, the new LAPAQ score is on a scale from 0 to 23. It was found highly predictive for mortality and the reliability of the new score was moderate to good.^28^ More details on the new LAPAQ score can be found in the e-Appendix.

### Assessment of outcomes

Experienced and trained study physicians or nurses assessed patients’ prevalent comorbidities at the baseline interview. First, patients completed open-ended questions regarding their comorbidities. Then, experienced and trained study personnel compared the comorbidities reported by patients with their list of medications (in Switzerland also their medical charts). Mismatches between patients’ responses and their list of medications (or charts) were resolved by clarification with patients’ general practitioners. Depression and anxiety were measured using the Hospital Anxiety and Depression Scale questionnaire. We considered patients to have clinically relevant levels of anxiety or depression if their score was 11 points or higher to ensure the “caseness” for depression and anxiety.^29^ Incident comorbidity profiles were updated every 6 months via postal questionnaires/phone interviews and face to face contacts (at two and 4 years of follow-up). We then created categories of these comorbidities.

### Statistical analysis

We performed multinomial logistic regression (STATA, version 13.1, College Station, TX, USA) as a close approximation to competing risk discrete time proportional hazards model to assess the association of PA with incidence of comorbidity while treating death (a competing event) as a separate category.^30, 31^ We chose this model instead of other survival models because comorbidity data were updated at 6-monthly occasions. Firstly, we examined the risk of developing a new (or an additional) comorbidity in all patients. Then, we examined the risk of developing each category of comorbidity separately. We identified the subjects who were free of the comorbidity at baseline and who had at least one additional follow-up visit. We defined survival time as the time to the first visit on which the occurrence of the comorbidity was identified. If no occurrence of comorbidity was identified, survival time was defined as the time to the last follow-up visit or, for those who died during study period, the time to the visit where the patient was reported dead. The relative-risk ratios estimated from multinomial logistic regression models were interpreted as hazard ratios. An example of how we implemented this model is provided in the e-Appendix.

We adjusted for potential confounders in the multivariable analyses, which included age, sex, body mass index (BMI), smoking pack-years, and forced expiratory volume in one second in liters (FEV1). Because that the induction time between exposure (PA) and outcomes (development of comorbidities) may be years or decades and that PA may already be influenced by some comorbidities before comorbidities are reported, we decided to use the baseline LAPAQ scores to assure the temporal relationship to comorbidities. We did sensitivity analyses using average of the values over time (from baseline to the last visit before the patient had the event or was censored) for PA and two covariates (BMI and FEV1), for which we had multiple assessments in our cohort. We recoded the independent variable LAPAQ score by dividing the score by 2.5 (the MET for walking outside) to aid interpretation of the hazard ratios, which estimate the increase in the risks for a 2.5-point increase in the LAPAQ score.

## Conclusions

Our investigation of the association of PA with comorbidity in COPD highlighted that patients with high levels of PA may be less likely to develop depression or anxiety over time. We observed modest, but plausible associations of PA with comorbidities such as cardiovascular, endocrine, musculoskeletal, malignant, and infectious diseases, while low numbers of incident morbidity yielded wide CIs. The results of our study imply that rehabilitation and PA promotion programs may not only be important to increase exercise capacity and levels of PA but also can be an approach for testing to lower the risk for incident depression and anxiety in patients with COPD.

## Electronic supplementary material

e-appendix
