# Supplementary material for: Physical activity and risk of comorbidities in patients with chronic obstructive pulmonary disease: a cohort study
Source: NPJ Prim Care Respir Med. 2017 May 18;27:36. doi: 10.1038/s41533-017-0034-x (PMC5437069; doi:10.1038/s41533-017-0034-x)
Supplement: Supplementary file 1 — e-appendix [file 41533_2017_34_MOESM1_ESM.docx]

**Supplementary Material**

Physical activity and risk of comorbidities in patients with chronic obstructive pulmonary disease: a cohort study

Tsung Yu, Gerben ter Riet, Milo A. Puhan, Anja Frei

**Assessment of physical activity**

We used the Longitudinal Ageing Study Amsterdam Physical Activity Questionnaire (LAPAQ) to assess patients’ physical activity levels. At baseline and follow-up visits patients were asked to indicate the frequency and duration of the 6 types of physical activity (walking outside, bicycling, gardening, sport activities, light and heavy household activities) they had performed during the previous two weeks. A LAPAQ score that integrates the time spent on each physical activity (minutes per day in the previous two weeks) and the intensity of each physical activity, informed by the metabolic equivalent tasks (METs), can be generated. However, we decided to construct a modified LAPAQ score without including the information on frequency and duration. We relied on patients’ responses to the types of physical activity they did and assigned weights to each physical activity according to the METs: 2.5 for outside walking, 4.0 for bicycling, 4.0 for gardening, 6.0 for sports, 2.5 for light household activities and 4.0 for heavy household activities. For example, if a patient indicated that s/he had walked outside and played sports during the past 2 weeks, her/his LAPAQ score would be 8.5 (2.5 + 6.0).

We decided to use the modified LAPAQ score for our analysis because of the following findings. First, at baseline 20% of the responses to the frequency or duration of physical activity were incomplete and some responses were suspected to be implausible (e.g., some patients responded that they had performed more than 12 hours of sports every day for the past two weeks). Second, we calculated the Pearson correlation coefficients between the original LAPAQ score (or the modified score) and patients’ performance on sit-to-stand test, an assessment of exercise capacity. We expected that physical activity is positively correlated with exercise capacity. The correlation was stronger for the modified score versus the original score (0.42 vs 0.16). Third, we calculated the test-retest reliability expressed in intraclass correlation coefficient (ICC) of the original LAPAQ score and the modified score. We assumed a patient’s level of physical activity did not change between the baseline visit and visit 1. The value of ICC was larger for the modified score compared to the original score (0.71 vs 0.43). Finally, we checked whether the new LAPAQ score was predictive for mortality. We found a higher score was associated with a lower risk for mortality (hazard ratio 0.76 per 2.5-point increase in the LAPAQ score; confidence interval 0.68 – 0.85; p<0.001).

**An example of multinomial logistic regression analysis**

Here we provide an example of how we implemented multinomial logistic regression analysis that approximates competing risk discrete time proportional hazards model. Suppose that our dataset has the following variables:

| **Variables** |  |
| --- | --- |
| ***id*** | Unique patient identifier |
| ***visit*** | Study visit number. Visit 1 is 6 months from baseline and the next semi-annual visits are numbered sequentially. Individuals can contribute to ≤10 visits in the dataset. |
| ***log_visit*** | Natural logarithm of visit |
| ***event_cvd_death*** | An indicator variable  = 2 if the patient reported a new comorbidity (here for example cardiovascular disease). = 1 if the patient died during the time interval between the previous visit and the current visit (intended). = 0 if otherwise. |
| ***pa*** | Physical activity summary score |
| ***bmi*** | Body mass index |

We then organized the dataset so that each patient contributed X rows in the dataset, where X is the number of visits the patient was at risk of different types of events. We have two types of events: cardiovascular disease and death. Here log (visit) is chosen as the functional form for the baseline hazard function. Below are entries from the first five patients in the dataset (a mock-up dataset):

| ***id*** | ***visit*** | ***log_visit*** | ***event_cvd_death*** | ***pa*** | ***bmi*** |
| --- | --- | --- | --- | --- | --- |
| 1 | 1 | 0.00 | 0 | 15 | 20 |
| 1 | 2 | 0.69 | 0 | 15 | 20 |
| 1 | 3 | 1.10 | 0 | 15 | 20 |
| 2 | 1 | 0.00 | 0 | 21 | 21 |
| 2 | 2 | 0.69 | 0 | 21 | 21 |
| 2 | 3 | 1.10 | 0 | 21 | 21 |
| 2 | 4 | 1.39 | 0 | 21 | 21 |
| 2 | 5 | 1.61 | 2 | 21 | 21 |
| 3 | 1 | 0.00 | 0 | 10 | 19 |
| 3 | 2 | 0.69 | 0 | 10 | 19 |
| 3 | 3 | 1.10 | 0 | 10 | 19 |
| 3 | 4 | 1.39 | 0 | 10 | 19 |
| 3 | 5 | 1.61 | 0 | 10 | 19 |
| 3 | 6 | 1.79 | 0 | 10 | 19 |
| 3 | 7 | 1.95 | 0 | 10 | 19 |
| 3 | 8 | 2.08 | 0 | 10 | 19 |
| 3 | 9 | 2.20 | 0 | 10 | 19 |
| 3 | 10 | 2.30 | 0 | 10 | 19 |
| 4 | 1 | 0.00 | 0 | 4 | 26 |
| 4 | 2 | 0.69 | 2 | 4 | 26 |
| 5 | 1 | 0.00 | 0 | 3 | 25 |
| 5 | 2 | 0.69 | 0 | 3 | 25 |
| 5 | 3 | 1.10 | 1 | 3 | 25 |

We used the following command to fit a multinomial logistic regression model:

***mlogit event_cvd_death pa bmi log_visit, rrr baseoutcome(0)***

The exposure of interest is physical activity, and the outcome is cardiovascular disease, while considering death to be a competing event. We adjusted for body mass index when estimating the model. Here we interpret the relative-risk ratios estimated in the model as hazard ratios [1, 2].

**Results comparing main versus sensitivity analysis**

|  | **Main analysis** | **Sensitivity analysis** |
| --- | --- | --- |
|  | **Physical activity: baseline value (LAPAQ score)** | **Physical activity: average of the LAPAQ scores over time** |
| **Comorbidity** | **Adjusted hazard ratio (95% confidence interval; P value)** | |
| An additional comorbidity | 0.95 (0.88 – 1.01; 0.12) | 0.93 (0.86 – 1.01; 0.08) |
| Cardiovascular diseases | 0.97 (0.80 – 1.18; 0.77) | 0.99 (0.80 – 1.23; 0.95) |
| Neurological disorders | 1.03 (0.85 – 1.25; 0.73) | 1.13 (0.91 – 1.41; 0.26) |
| Endocrine disorders | 0.92 (0.60 – 1.41; 0.71) | 0.88 (0.52 – 1.49; 0.64) |
| Musculoskeletal disorders | 0.88 (0.74 – 1.04; 0.12) | 0.83 (0.69 – 1.00; 0.06) |
| Mental disorders | 0.92 (0.83 – 1.02; 0.13) | 0.91 (0.81 – 1.03; 0.13) |
| Cancers | 0.86 (0.65 – 1.13; 0.27) | 0.86 (0.64 – 1.17; 0.35) |
| Infectious diseases | 0.85 (0.69 – 1.03; 0.09) | 0.83 (0.67 – 1.03; 0.10) |
| Depression | 0.85 (0.75 – 0.95; 0.005) | 0.83 (0.73 – 0.94; 0.004) |
| Anxiety | 0.89 (0.79 – 1.00; 0.045) | 0.90 (0.80 – 1.03; 0.12) |

**Abbreviation.** LAPAQ = Longitudinal Ageing Study Amsterdam Physical Activity Questionnaire

**Note.**

1. We adjusted for age, sex, smoking history, body mass index and forced expiratory volume in 1 second in the model.
2. The average of the LAPAQ scores over time was calculated using values from baseline visit to the last visit before the patient had the event or was censored.
3. The unit of physical activity is 2.5-point increase in the LAPAQ score.

**References**

1. Allison, P.D., *Discrete-time methods for the analysis of event histories.* Sociological

methodology, 1982. **13**(1): p. 61-98.

2. Kimber, J., et al., *Survival and cessation in injecting drug users: prospective observational study of outcomes and effect of opiate substitution treatment.* BMJ, 2010. **341**: p. c3172.
